# Pulmonary Suffusion Refinements for Primary and Secondary Malignancies: Preliminary Analyses of Phase I Safety and Drug Delivery Data †

**DOI:** 10.3390/cancers17172880

**Published:** 2025-09-02

**Authors:** Todd Demmy, Samah Abdelhady, Garin Tomaszewski, Michael Petroziello, Omar Hasan, Mark Hennon, Elisabeth Dexter, Deepak Vadehra, Ajay Gupta, Anne Grand‘Maison, Grace Dy, Sai Yendamuri

**Affiliations:** 1 Department of Thoracic Surgery, Roswell Park Comprehensive Cancer Center, Elm & Carlton Strs., Buffalo, NY 14263, USA; mark.hennon@roswellpark.org (M.H.); elisabeth.dexter@roswellpark.org (E.D.); sai.yendamuri@roswellpark.org (S.Y.); 2 Department of Pediatric Hematology and Oncology, Roswell Park Comprehensive Cancer Center, Elm & Carlton Strs., Buffalo, NY 14263, USA; samah.abdelhady@roswellpark.org (S.A.); ajay.gupta@roswellpark.org (A.G.); 3 Department of Radiology, Roswell Park Comprehensive Cancer Center, Elm & Carlton Strs., Buffalo, NY 14263, USA; garin.tomaszewski@roswellpark.org (G.T.); michael.petroziello@roswellpark.org (M.P.); omar.hasan@roswellpark.org (O.H.); 4 Department of Medical Oncology, Roswell Park Comprehensive Cancer Center, Elm & Carlton Strs., Buffalo, NY 14263, USA; deepak.vadehra@roswellpark.org (D.V.); grace.dy@roswellpark.org (G.D.); 5 Center for Cancer and Oncology Care, Erie County Medical Center, Buffalo, NY 14215, USA; agrandmais@ecmc.edu

**Keywords:** pulmonary suffusion, metastasectomy, DNA-damaging agents, colorectal cancer, soft tissue sarcoma

## Abstract

**Objectives**: We sought to document interim methodologic improvements and preliminary results for pulmonary suffusion. **Methods**: A Phase I/II trial of thoracoscopic lung suffusion for resectable sarcoma and colorectal carcinoma metastases followed a pilot study on oligometastatic lung malignancy at a comprehensive cancer center. Primary-specific chemotherapy doses (cisplatin, oxaliplatin, doxorubicin, or gemcitabine) suffused unilaterally for 30 min were escalated to amplify regional deliveries three-fold. Drug delivery was measured with tissue, blood samples, and ^99^Tc; pulmonary function tests and clinical adverse events (AEs) assessed safety and tolerance. **Results**: From 2008–2025, 31 ECOG 0–2 patients (10 male) aged 33–75 years had unilateral lung suffusion (16 right, 14 left, 1 aborted, and 8 sides selected randomly). Vascular occlusion intolerance was immediate or delayed (25 min) in two cases. Two catheter-positioning grade 3 AEs occurred: hypotension with troponin leak (1) and atrial fibrillation (1). Patients averaged 1.3 ± 1.2 metastasectomies (17 sub-lobar, 8 lobar resections, and 2 intentional open cytoreductive metastasectomies). Hospitalizations were brief (1–4 days) except for 6–7 day stays in the only two open cases and one doxorubicin (grade 4 hypoxic respiratory failure) case. Ninety-day survival was 100%, and the Phase I delivery goal of 12.75 mg/m^2^ 65 (15% systemic) was achieved for oxaliplatin. Lung function was preserved according to ^99^Tc differentials within 6.1 ± 7.1% of the predicted reductions at 30 days. Sampling delays, tracer discordances, and atypical pharmacokinetics reduced tissue drug detections. Recent pulmonary artery snaring cases (two) demonstrated in-flow control more stable than that of balloon occlusions. **Conclusions**: Suffusion for metastatic malignancies appears safe and warrants further investigation.

## Simple Summary

Lung malignancy (originating there or from other sites) causes significant sickness and death, with treatment options often limited by drug toxicities or tumor locations preventing other therapies like surgery or irradiation. Suffusion is a novel treatment delivering chemotherapy directly into the closed-off lung’s circulation for 30 min to enhance its effect on lung cancer or metastases while avoiding systemic toxicity. In this trial, we gave chemotherapy to one lung followed by tumor observation or the surgical removal of lung metastases from other cancers. Suffusion was intended to suppress the growth of microscopic tumors and in most cases was performed minimally invasively to reduce risks. Because of substantial learned improvements in suffusion technique, we present this preliminary safety report.

## 1. Introduction

The second most common cause of cancer-related deaths worldwide is primary lung malignancy [1]. Pulmonary metastases from other malignancies (like colorectal and sarcoma) also generate considerable morbidity and mortality [2]. Surgery and other local therapies like stereotactic body radiation therapy (SBRT) are offered to cure these conditions; however, many patients have tumors too extensive for local therapies [3,4,5,6]. Even lung tumors eligible for removal or ablation are prone to recurrences from micrometastatic minimal residual disease not detectable on standard clinical imaging [7,8].

To provide additional options for minimal residual oncologic disease in efforts to improve survival, we and others have devised regional strategies for cancer treatment. Recirculating pump-based therapies are technically complex and quite invasive but attempt to deliver consistent, high concentrations of chemotherapy from a reservoir [9,10,11,12,13]. Less invasive techniques use inhalation [14], bronchial artery infusions [15], chemoembolization [16,17], and direct tumor injections [18], but each of these have their own challenges [19].

In 2008, we initiated a clinical program for a simplified, minimally invasive, stop–flow local drug amplification approach and called it “suffusion” to differentiate it from previous endeavors [20]. Simple direct pulmonary artery (PA) infusion without restricting flow is no more effective than systemic administration because of the rapid dilution [21]. In 1960, Smyth and Blades used a PA occlusion strategy to amplify regional delivery of nitrogen mustard and achieved local effects in 2 of 3 patients, but 75% of the drug leaked systemically within 10 min [22]. To enhance this, suffusion provides simultaneous venous occlusion and achieves 75% retention of escalating doses of various chemotherapy drugs in the lung for 30 min [23]. One of those drugs (Oxaliplatin) completed Phase I and is currently in Phase II. Herein, we describe the technical challenges and solutions, potential toxicities, and participant selection changes over the past 18 years so that others may be able to replicate this work safely.

## 2. Materials and Methods

### 2.1. Overall Study Design—General

We sought to determine the safety and dose-limiting toxicity (DLT) of the regional drug chemotherapy method termed suffusion for oligometastatic lung cancer and later pulmonary metastases. Eligible patients received suffusion to one lung with laterality determined by randomized allocation when metastatic disease was bilateral.

### 2.2. Evolution of Eligibility Criteria and Design

**Figure 1:**
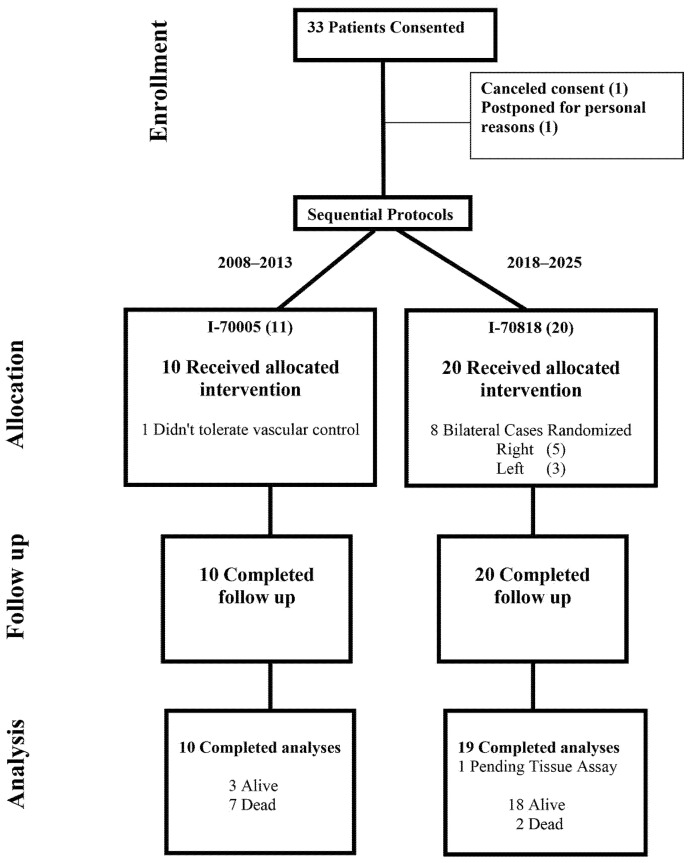
Consort diagram. The illustration demonstrates the accrual and management of participants over the duration of the trial.

Accrual and eligibility for suffusion protocols have evolved as required by changes that occurred in clinical medicine such as the expansion of immunotherapy. The trial (I-70005) started as a pilot study for feasibility and safety in 2007 for oligometastatic non-small-cell lung cancer patients with suffusion performed on the side of the primary tumor. The goal was to not delay systemic therapy (planned 2 weeks following suffusion for lung cancer patients). Favorable safety findings and insurance coverage for standard-of-care operations enabled cohort expansion to locally advanced lung cancer and pulmonary metastasectomy for other primaries. The eligibility and design changed (I-70818) in 2018 to Phase I/II for sarcoma patients (either soft tissue sarcoma or osteosarcoma) since this group typically has chemotherapy-resistant tumors (Figure 1). Because of physician referrals and participant interest, the cohort was expanded to include colorectal carcinoma (CRC) patients in 2022.

#### Eligibility Requirements

The performance status (PS) requirement was initially ECOG (Eastern Cooperative Oncology Group) 0–1, but this was expanded to include ECOG 2 in 2018. Metastatic patients were required to have stable primary tumors and no other extrathoracic disease. Patients with brain metastases were ineligible for the trial. Patients with human immunodeficiency virus (HIV) were excluded from I-70005 but are allowed currently if their infection is well-controlled.

Suffusion for pulmonary metastases occurred after standard care to control the primary disease and/or extra-thoracic metastases. For patients needing systemic chemotherapy including investigational drugs, washout periods of 4–6 weeks were required depending on the individual drug half-life. Diagnoses were confirmed before suffusion by histopathology/cytology or by intraoperative biopsy

### 2.3. Phase Changes Including the Dose Escalation Rationale

Excluding drugs with accelerated first-pass clearance, about 5% of a typical systemic chemotherapy intravenous infusion is delivered to each lung. This is estimated based on the relative systemic to pulmonary blood volume distribution under normal physiologic conditions [19,24]. Accordingly, 5% of a normal systemic dose (NSD, see below) was used as the first level for cisplatin (and oxaliplatin). Five times (25%) this relative dose of cisplatin was tolerated well in preclinical experimentation that also led to the recommended starting doses of doxorubicin and gemcitabine at 7.5% [25]. While higher doses may be safe, we targeted three-fold local augmentations (15% systemic) for this initial work. Cisplatin (NSD = 75 mg/m^2^) was the only drug used for the first 10 patients (Table 1) with various pathologies [26,27]. Doxorubicin (NSD = 75 mg/m^2^) was preferred for soft-tissue sarcomas (4) and oxaliplatin (NSD = 85 mg/m^2^) for colorectal cancer (14) [28,29]. Gemcitabine (NSD = 1000 mg/m^2^) (2) served as a backup for CRC and sarcoma participants unable to tolerate first-line drugs because of problems like hypersensitivity reactions [30]. Because of some evidence of a differential effect in I-70005, the design was transitioned to Phase I/II to realize a maximum tolerated dose (MTD) for each drug and (Phase II) suppression of emergence of minimal residual disease after metastasectomy.

### 2.4. Objective Eligibility and Suffusion Toxicity/Effect Assessment

Pulmonary function tests, thoracic imaging, symptom scoring, and lung differential scans were measured before and at timepoints up to 30 days after suffusion. Forced expiratory volume in 1 s (FEV1), diffusing capacity of carbon monoxide (DLCO), vital capacity (VC), and 6 min walk test (6-MWT) requirements were set to ≥50% of that predicted. Modified Borg dyspnea scale (<5) [31], ambulatory and resting O_2_ saturations >88% on room air, and complete blood count (CBC) and comprehensive metabolic panel (CMP) data demonstrating sufficient baseline hematologic and organ function were required. These tests as well as Technetium-99 macroaggregated albumin (^99^Tc) lung differential scans were used to document baseline functions, hematologic toxicities, and relative suffusion damages at 30 days. Chest roentgenograms (CXR) performed on days 0, 1, 3, 7, and 30 detected immediate pulmonary toxicities. Dyspnea scales (except day 0) and 6-MWT (days 7 and 30) were used to evaluate post-suffusion performance. Chest computed tomography (CT) scans of primary tumors or metastases at registration and during regular follow up were used to evaluate residual disease or recurrence. Guidelines for evaluation of measurable disease response and progression were followed in accordance with RECIST 1.1 (version 1.1).

#### 2.4.1. Estimation of Post-Surgery Predicted Lung Function

To differentiate whether lung damage was from surgery or chemotherapy effects, we estimated predicted pulmonary function tests (PFTs) and lung differentials based on removed segments:

(Total segments − removed segments)/Total segments × 100%.

To account for multiple wedge resection patients, we used a similar formula to predict residual function by subtracting the aggregate resected pathologic lung weights from published tables of normal estimated total right and left lung weights based on patient sex and body size [32].

#### 2.4.2. Pharmacological Measurements of Suffusion Delivery

Because it was impractical to obtain direct lung samples while supine during lung ventilation using VATS incisions, bilateral bronchial lavage and PA blood sampling was performed about 2–3 min before vascular release for chemotherapy detection. ^99^Tc tracer scanning helped demonstrate drug delivery because terminal suffusion lung samples were impractical to obtain. Upper and lower lobe lung samples for drug levels were taken as soon as possible after restarting thoracoscopy. Systemic blood samples at 15 and 60 min after vascular release tracked drug leakage.

### 2.5. Suffusion and Metastasectomy Techniques

**Figure 2:**
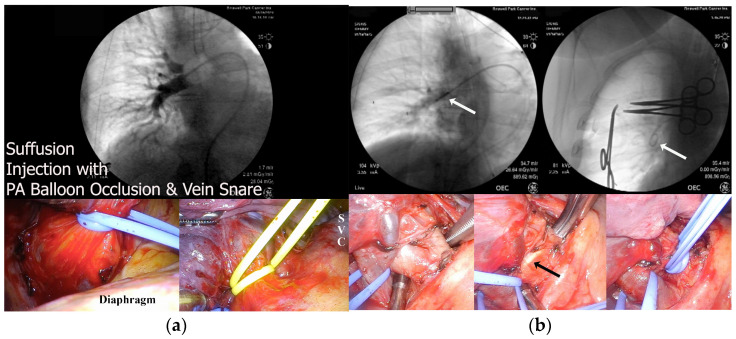
Right lung suffusion control examples. (**a**) Standard vascular control using a 9F pulmonary artery balloon catheter placed in the operating room by the interventional radiologist, with thoracoscopic single snare loops on pulmonary veins shown (blue, inferior; yellow, superior). Notice the balloon encroaching fluoroscopically onto the upper lobe segmental arterial branches. This proximal location is necessary for suffusing the entire lung but makes the balloon vulnerable to dislodgement. (**b**) Enhanced vascular control that uses the same venous strategy as above but with a 6F cardiac pigtail catheter (arrow) replacing the balloon catheter. This catheter can be seen through the arterial wall (arrow). A doubled snare on the main right pulmonary artery encircles the catheter to prevent leakage. The right fluoroscopic panel shows the hemostats on the chest wall that maintain snare traction.

The technique has been described before [26]. In brief, double-lumen endotracheal tube general anesthesia was administered. The patient was rotated to lateral decubitus position, ipsilateral lung collapse was induced, and the pulmonary veins were dissected and double-looped (silicone vessel tapes) for later snaring and occlusion when retracted. The loose (non-occlusive) snare ends were sutured just beneath the most anterior VATS (video-assisted thoracoscopic surgery) wound sites that had temporary closures, and then, the lung was reinflated. The patient was placed supine for PA catheter placement (usually right femoral vein) using c-arm fluoroscopy (General Electric Healthcare OEC 9900, Chicago, IL, USA; Figure 2a). Observation of wedge pressures ensured PA occlusion. Vessel loops were retrieved through re-prepped VATS incisions and retracted, creating an occlusive snare effect intracorporeally. Retraction was maintained by hemostat clamps large enough to bridge both sides of the VATS wounds. Omnipaque™ 240 (General Electric Healthcare, Chicago, IL, USA) or similar radiographic contrast confirmed correct balloon catheter location. Before chemotherapy infusion, patients received 5000 IU intravenous heparin, dexamethasone, and ondansetron. Catheter position was verified fluoroscopically approximately every 5 min, and PA pressures were monitored continuously (Supplementary Video S1). The suffusion sequence was (1) ipsilateral ventilation cessation with PA inflow occlusion by balloon inflation and venous occlusion by snaring, (2) repetitive blood withdraw from PA and immediate venous return, (3) lung reventilation, (4) 1–4 mCi ^99^Tc (<1 mL vehicle) injection into the ipsilateral PA, (5) injection of chemotherapy (diluted to 1 mL/kg), (6) dwelling for 30 min, and (7) suffusion termination by balloon deflation and unsnaring of the pulmonary veins. The slick silicone snare tapes were extracted completely by pulling on one end of the loop (while supine), thereby ensuring that the veins were open immediately after the suffusion, and the PA catheter was removed before repositioning the patient. After the patient was re-positioned to the lateral decubitus position, metastasectomy (occasionally requiring formal lung resection) was performed with tumor and lung tissue sent for pathologic evaluation and correlative science storage. For bilateral disease patients, contralateral metastasectomy (without suffusion) was performed under that same anesthetic or staged (within 90 days) based on clinical tolerability. Two patients with numerous metastases had staged thoracotomies with stapled and enucleated resections and had planned post-resection ablative techniques for residual missed lesions.

### 2.6. Required Technical Adjustments over the Course of Study

#### 2.6.1. Occlusion Catheter Usage

The Arndt™ 9 French (Cook Medical, Bloomington, IN, USA, off-label use) bronchial occlusion balloon performed best because of the optimal length, low-pressure air-filled balloon, side and tip infusion/drainage holes, and stiff durometer that made positioning difficult but also resisted dislodgement. Generally, once the desired PA was cannulated, an exchange catheter was needed to place a stiff Amplatz wire (Boston Scientific, Marlborough, MA, USA) for introduction of the Arndt catheter. Importantly, other large balloon occlusion products like the Cook Coda™ LP catheter (Cook Medical, Bloomington, IN, USA) and the Boston Scientific Equalizer™ (Boston Scientific, Marlborough, MA, USA) were easier to place but could not be aspirated due to lack of a side drainage lumen, and these lacked sufficient durometer to remain stable in the proximal PA. For our last two cases, we used 6F cardiac (non-balloon) pigtail catheter 10 (Performa^®^, Merit Medical, South Jordan, UT, USA) that was easier to place and allowed easier procurement of lung samples during suffusion (Supplementary Video S2). A double snare loop (Figure 2b) placed surgically around the proximal PA occludes the arterial inflow by cinching the vessel around the catheter. This catheter can be placed at the start of the operation and maintained with a heparin infusion, eliminating the need to reposition the patient, provided that c-arm fluoroscopy is performed with a cross-table technique.

#### 2.6.2. Radiotracer Concordance

We initiated ^99^Tc scanning at Case 7 to demonstrate drug delivery by correlative means since terminal suffusion lung samples were impractical to obtain. Tracer was given once after instilling the chemotherapy and for the rest of the cases as the first item infused. Dynamic scanning was performed once with the portable Ergo™ (Digirad, Suwanee, GA, USA) system, and the remainder of the patients were transported to nuclear medicine for scanning at the completion of their operation.

### 2.7. Statistical Plan

The primary endpoint for the Phase I portion was any grade 3 or higher local or systemic toxicities based on NCI CTCAE v5.0 (Common Terminology Criteria for Adverse Events version 5.0); these were considered for the analysis of the dose-limiting toxicity (DLT). The DLT for this study was a composite of toxicities related to the suffusion procedure technique and toxicity (largely ipsilateral pulmonary) caused by the chemotherapeutic agent. Accrual was halted for either, and remedies were added by protocol amendments as necessary. Phase I trials followed a 3 + 3 design with 3 patients starting the new dose level. After a DLT, another 3 patients were added. When 0 or 1 DLTs were obtained for six cases, the dose was considered safe and could be increased to the next dose level. The MTD in Phase I was considered the starting dose for Phase II studies.

The Phase II statistical analysis for the potential efficacy of suffusion was based on a maximal sample size of 50 patients to detect an observed 20% reduction in metastatic recurrence in 2 years in the suffused side compared with the contralateral. The expected rate was adjusted based on the proportion of primary tumor types accrued to this trial and their published rates of recurrence.

Data were analyzed using Minitab Statistical 22 (State College, PA, USA) and GraphPad Prism Version 10 (Boston, MA, USA). Continuous variable data are presented as means ± std. dev. Groups were compared using two tailed *t*-tests (unless noted otherwise) and analysis of variance (ANOVA) or Wilcoxon and Kruskal–Wallis tests for comparing differences in non-parametrically distributed data. For categorial data, the Chi-squared test was used to analyze the frequency of observations. Significance was considered at a probability < 5%.

## 3. Results

Given the rapid accrual for CRC, the oxaliplatin Phase I cohort completed its highest planned dose level and started Phase II. Phase I cohorts for cisplatin, doxorubicin, and gemcitabine (MTDs not yet determined) remain open, but analyses of these data will be discussed for the purpose of describing overall suffusion safety and important improvements with the suffusion technique.

### 3.1. General Demographics

Between February 2008 and January 2025, suffusion was attempted on thirty-one ECOG 0–2 patients, and only one (Case 9) did not tolerate initial vascular control, which prevented drug delivery (Figure 1). In total, 30 patients received suffusion with one of four chemotherapy drugs. (Table 1). Supplementary Table S1 lists values for the oxaliplatin cohort only, as it is the only drug that has a complete Phase I.

Participant age was 55 ± 12 (range 33–75), and 20 participants (67%) were female. There were 16 right and 14 left suffusions, and 8 with bilateral metastases underwent randomization (5 right and 3 left). Twenty-eight cases were approached thoracoscopically with an average of 1.3 ± 1.2 metastases resected. Two intended thoracotomies (one after preliminary VATS for vein control and another where the suffusion was performed laterally with a snare around the PA catheter) were used to resect many lesions (34 and 8). Patients underwent 14 sub-lobar and 8 lobar resections, surgery time averaged 408 ± 110 min (Table 2), and the estimated blood loss was 80 ± 80 mL. The catheter insertion time was shorter with pigtail placement (the last two cases had times of 12 and 35 min); however, more time was needed to encircle the main right PA.

### 3.2. Early Clinical Outcomes Including Catheter and Possible Drug-Related Adverse Events (AEs)

Procedures were generally well-tolerated with no operative, hospital, or post-operative mortalities (by 30 days). For VATS patients, hospital stay (Table 2) was brief (1–3 days) except for a doxorubicin DLT patient discharged on day 7 and another case medically cleared for discharge (chest drain out) on day 1 but delayed to day 4 because of social issues. The thoracotomy patients (above) with extensive chemotherapy pretreatment and multiple metastasectomies were discharged on days 6 and 7, respectively, without suffusion-related adverse events or other major complications. Minor events like pain common with surgical recovery (four per patient) did not increase hospital stay. See Supplementary Table S2 for details of these and other AEs attributed to metastasectomy rather than suffusion. Two grade 3 (Gr3) AEs were attributed to catheter-related placement/positioning. The first (Case 6, cisplatin, 7.5% systemic) developed hypotension 25 min into suffusion that recovered immediately following vascular occlusion release. A mild troponin elevation occurred the next day. The patient was monitored in the ICU, extubated after 6 h, and discharged on day 3 (needed 3 L/min supplemental oxygen stopped postoperative day 6). Three additional patients without DLTs allowed escalation to 10% of the systemic dose that one patient tolerated. The second (Case 29, oxaliplatin, 15% systemic) had sustained partial main PA obstruction and transient atrial fibrillation (Gr3) from catheter dislodgement at minute 20. This was controlled by suffusion termination and intraoperative beta blockade without further sequalae. Neither patient had pulmonary infiltrates or systemic chemotherapy-related toxicity.

Patient 12 (doxorubicin, 5.625 mg/m^2^, 7.5% systemic) developed respiratory failure (Gr4) from ipsilateral pulmonary edema on day 2. She received steroids, ICU observation (3 days), and supplemental oxygen (24 h of high flow) before transitioning to room air by day 5. Post-suffusion ipsilateral lower lobectomy, no preparatory dexamethasone (reported hypersensitivity), and prolonged PA cannulation (150 min) may have contributed to this AE. Reduced doxorubicin doses for the next three patients (3.75%) yielded no adverse events, which allows dose re-escalation in the upcoming eligible patient.

#### Early (30-Day) Outcomes

There were no systemic chemotherapy toxicities from suffused drugs (as was shown in patients’ blood chemistry and blood counts), and resumption of standard anti-neoplastic treatments resumed without delays caused by participation. Lung cancer patients started chemotherapy within 2 weeks (expected per I-70005 protocol guidelines). Other patients with different primary cancers received recommended treatment according to standard-of-care guidelines when required by the recurrence of disease or ongoing maintenance therapy.

**Figure 3:**
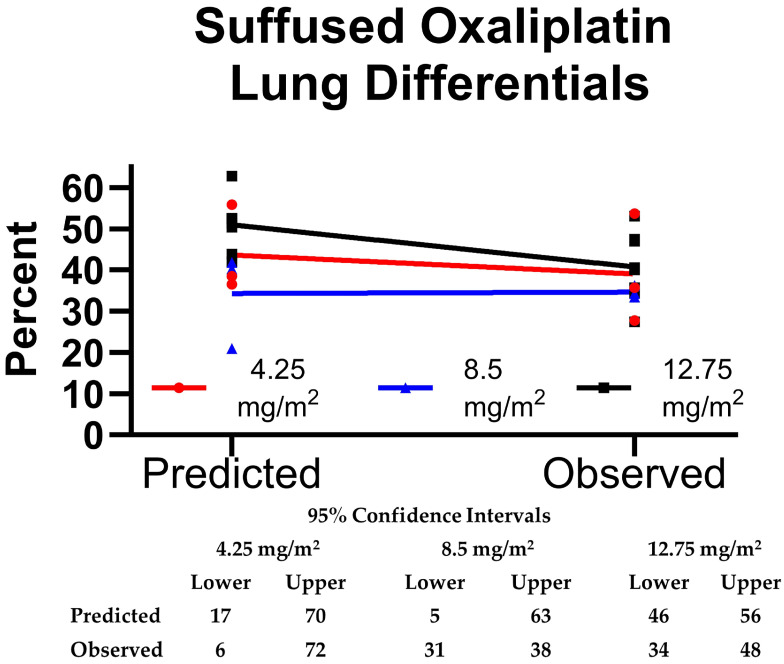
Comparison of predicted change in split lung function versus that observed at 30 days. Only the 12.75 mg/m^2^ group change was significant.

By using the calculation of the remaining lung function by number of segments removed, a reasonable estimate was achieved, but using the weight of lung removed was more accurate. For all cases, mean 30-day FEV1 and DLCO were predicted to fall 12% from pre-suffusion values (in Table 1, also Supplementary Table S3), with post-observed values of 81.6 ± 14.2 and 76.8 ± 21.1 (−5.8 and 1.8% variances), respectively. Before and after, 6-MWT tracked closely with DLCO (73.3 ± 21.7%). Using similar methodologies, the observed split lung function on the suffused side was 38.8 ± 10.5%, which was −6.1% less than expected. The oxaliplatin cohort is plotted in Figure 3. While the lower dose levels had little effect, the 12.75 mg/m^2^ group did demonstrate a 10.2% greater reduction in differential contribution than predicted (*p* = 0.003). Supplementary Figure S1 connects the predicted and observed points in Figure 3 and demonstrates the outlier effects on the differential from a lobectomy and a 34-lesion metastasectomy case.

### 3.3. Tracer Indicator for the Desired Drug Distribution

Despite radiologic dye confirmation pre-suffusion, patients’ contralateral (8) and balanced (1) tracer distributions were considered discordant. See the Section 4 for possible reasons for discordance. Discordance did not have noticeable effects on lung function or tumor response.

### 3.4. Blood and Tissue Drug Levels

**Figure 4:**
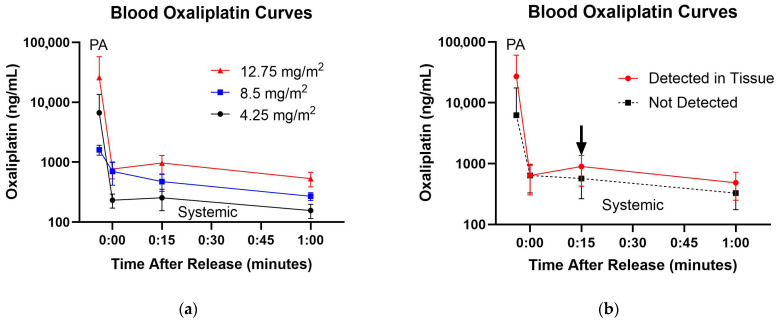
Blood levels of oxaliplatin. (**a**) Escalating doses of oxaliplatin demonstrate proportionate increases in pulmonary artery and systemic blood levels. Systemic drug levels were significantly different (*p* = 0.03, ANOVA, Supplementary Table S4). (**b**) Lung tissue oxaliplatin associated with higher concentrations in PA blood before balloon release and systemic rise (redistribution) at 15 min (indicated by arrow). Aggregate non-detection curves had lower peak PA levels without such rises.

Preclinical studies showed high drug residuals trapped in the PA and ipsilateral lung near the end of suffusion along with low systemic levels. After vascular release, a secondary peak in the circulating drug should occur unless metabolized (or tissue-bound as transpires with lower doses, Figure 4). In the oxaliplatin cohort, this expected preclinical pattern occurred in nine patients, with the post release “bump” observed in six patients (two cases were missing because of early suffusion termination). Detectable or quantifiable lung tissue drug levels were found in 17 patients (61% of total patients) and correlated inversely with relatively long washout times (39.6 ± 18.4 min). The two oxaliplatin cases sampled before washout (PA snaring) had the high levels seen preclinically (32,950 vs. 1523 ng/g).

When comparing all the post-release values, tissue drug detection was associated with 438 ng/mL higher blood levels (*p* = 0.105, 95% confidence −131 to 1007) with a significant 15-min post-suffusion systemic oxaliplatin bump indicated by the arrow on Figure 4b (+301 versus −49.5 ng/mL, *p* = 0.039, Kruskal–Wallis). For balloon occlusion cases, difficulty aspirating lung blood after 100 mL indicated better vascular control with more drug detection in cases with lower median aspirates (130 vs. 240 mL, *p* = 0.05, Kruskal–Wallis). ^99^Tc tracer concordance occurred in 5 of 6 bronchial washing samples where the drug was detectable. Lavage assays were otherwise missing (5), unquantifiable (15), or undetectable (4).

### 3.5. Longer Term Outcomes

We report final outcomes for I-70005 (pilot, previous interim report [26]) and intermediate outcomes for I-70818 pertaining to safety.

#### 3.5.1. Cisplatin—Suffusion to Augment Local Control in Patients Undergoing Systemic Therapy for Oligometastatic Lung Cancer

**Figure 5:**
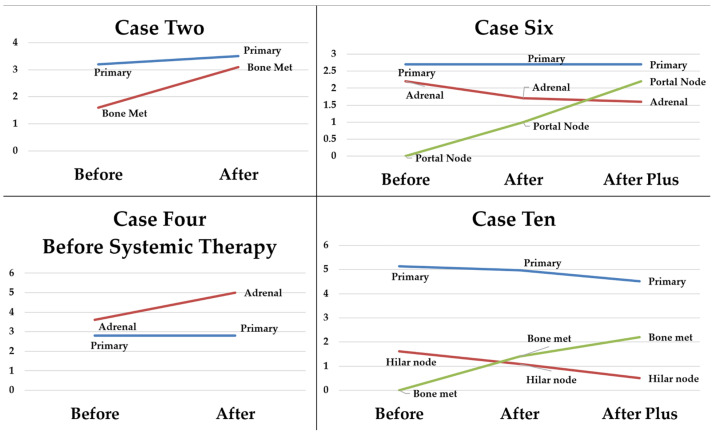
Computerized tomographic measurements (cm) of primary tumors and metastases. Before = within 1–4 weeks prior to suffusion. After = completion of 2nd chemotherapy cycle, generally 6–8 weeks except in Case 4, where imaging was performed before systemic therapy. After plus = an additional 2 months of therapy.

Seven patients underwent suffusion without resection of oligometastatic lung cancer (Table 3) in the era preceding routine chemoimmunotherapy. Cases 1 and 3 showed good initial responses but also had systemic therapy initiated after suffusion. Little change occurred with Case 7 that had low-grade multicentric minimally invasive adenocarcinoma. The remaining cases showed better control of tumors within the suffusion zones than distant metastases (Figure 5).

#### 3.5.2. Cisplatin—Suffusion to Suppress Micrometastatic Recurrence

Three patients underwent suffusion along with resection of metastases (Table 3) like I-70818. Case 8 with malignant peripheral nerve sheath tumor (MPNST) had rapid progression of disease within months of suffusion with no other obvious perioperative complications that might have accelerated recurrence. The other two cases did well and had no recurrence after prolonged follow up. Because of no osteosarcoma accruals, cisplatin had yet to be used in I-70818.

#### 3.5.3. Doxorubicin

For the doxorubicin sarcoma cohort, there were no accelerated progressions like that for Case 8. Two patients followed a typical course and succumbed to metastatic MPNST and uterine leiomyosarcoma 9 and 10 months following suffusion, respectively. The latter patient had a small brain metastasis and local recurrence based at the time of suffusion that could be identified retrospectively after. Two other patients are in remission 24 and 27 months following suffusion.

#### 3.5.4. Oxaliplatin and Gemcitabine

There have been no rapid CRC progressions or unusual recurrence patterns following oxaliplatin suffusion. Three of fourteen cases had incomplete metastasectomy resections, with one of these patients dying from an accidental overdose at 7 months. Twelve patients are alive 18 months (median, range 4–31) following suffusion. Six cases developed new metastases 4–16 months following suffusion, with three of these outside the suffusion zone. Eight cases remain in remission 4–24 months from suffusion. Both Gemcitabine patients are in complete remission at 18 months. Currently, too few events prevent comment on tumor suppression effects.

## 4. Discussion

This interim analysis of toxicity and vascular control enhancements demonstrates that pulmonary suffusion is a safe, promising regional therapy strategy worthy of additional investigation. Enough cases were completed to support suffusion as feasible and without evidence of lung injury caused by ischemia or vascular instrumentation. Hospital stays have been favorable compared to more complex regional strategies (2 vs. 7 days) [13]. Regarding efficacy, the cost of delivering chemotherapy in the operating theatre has so far prevented us from performing suffusion for unresectable metastases for which it is intended ultimately; however, there were examples of targeted intrathoracic effects on unresected patients in our pilot trial (Figure 5). One case (#6) showed a slight reduction in size of an adrenal metastasis which could also have received the drug by enhanced lymphatic delivery [33]. Furthermore, the mild reduction in observed lung differentials seen in higher dose levels (Figure 3) also demonstrates a regional delivery effect.

Direct PA snaring (performed preclinically) offered the advantages of reliable surgeon control, direct observation of vascular anatomy inclusive of upper lobe branches, and lower chance for balloon dislodgement. A disadvantage is tedious central hilar dissection that is potentially hazardous using minimally invasive techniques. Balloon PA occlusion was chosen because it was an established clinical technique, easily transitioned from the operating room, and did not require central dissection. However, this led to challenges with reliable balloon PA inflow occlusion and confirmation of drug delivery during the suffusion dwell phase that we attempted to study using ^99^Tc that was often discordant. It should be noted that this tracer was <2% of the injected total volume and was vulnerable to being displaced by the main chemotherapy bolus. Discordant ^99^Tc, despite catheter position confirmation by contrast injections, may occur from the following: failure to reventilate the target lung restricting vascular capacity, chemotherapy infusion “backwashing” the 1 mL tracer, insufficient balloon occlusion pressure, accelerated tracer degradation, or collateral systemic circulation causing PA flow reversal. Increased bronchial venous or lymphatic drainage may also accelerate suffusate leakage, causing the tracer to show up in the opposite lung. Tracer discordance and no post-suffusion systemic drug “bump” correlated with less tissue detection (although lungs also had prolonged washout before sampling). Ultimately, collateral systemic to pulmonary artery perfusion occurring normally or with pathologic conditions is unpredictable, making a single assessment using ^99^Tc impractical [34,35]. We intend to adopt the use of indocyanine green that is now very accessible clinically to allow repetitive assessments and potential quantification of collateral washout.

Peak suffusion lung sampling and reliable vascular control may be addressed by selective pigtail catheterization immediately after anesthesia induction (supine) and occluding all vessels thoracoscopically (posterolateral). We feel that long delays before lung sampling allows suffusion washout, making drug detection difficult. Because we can sample the lung parenchyma before washout in the lateral position, this technique yielded drug concentrations seen preclinically and currently represents our preferred vascular control method for suffusion [25]. This also allows us to phase out the use of unreliable bronchial washings to assess drug deliveries. Snaring may also be better for upper lobe delivery since its segmental artery is usually effaced by the PA balloon. Our initial concerns regarding generalizability are reduced because more thoracic surgeons now practice main PA dissection for snaring by VATS for hemorrhage prevention/control and pneumonectomy. Finally, combining PA snaring and balloon catheter usage is another option that was successful in a preclinical model designed to augment drug delivery to mediastinal nodes [36].

Direct PA cannulation downstream of the PA snare (replicating experimental work) could also reduce the need for transfemoral PA cannulation [25]. This would save a lot of operative time by allowing the entire suffusion process to be performed during a single patient positioning and making it easier to obtain lung samples while the suffusate is still dwelling. We also intend to adopt the use of Solid-Phase Micro Extraction fiber technology as is practiced at the University of Toronto, where surgeons employ coated fine acupuncture fibers to assess drug levels during regional therapy to avoid any loss of parenchyma [37,38].

Cypel and colleagues initiated in vivo Lung Perfusion with a similar patient eligibility and study design. Sufficient time has elapsed in their trial to detect possible suppression of minimal residual disease (27% vs. 53% in non-perfused lungs, N = 21), and we hope to reproduce their findings [13]. Their work is impressive since they intentionally created potential unfavorable bias by perfusing lungs with more metastases. A randomization strategy as we adopted may reveal an even greater differential effect, provided we can achieve similar local delivery augmentation.

Despite achieving our preclinical tissue drug levels, it is unclear whether suffusion will be as effective as longer-duration perfusion-based therapies. We hypothesize that there may be certain advantages to suffusion because of its relative simplicity and avoidance of blood pump and central vascular cannulation toxicities. Accelerated washout will likely occur in cases with robust collateral flow, but this could be addressed with supplemental doses. Longer intervals of suffusion are likely possible as well as topical heating and the use of new drugs including immune modulators. If regional therapies are effective for microscopic disease, there may also be benefits for macroscopic lesions that are unresectable because of their location or multitude. Thus, suffusion may be a tool for tumor cytoreduction and the selection of optimal second-line therapies by observing regional responses.

Surgical regional chemotherapeutics are encumbered by relatively high operating theatre costs, especially for patients who do not have medical insurance for accepted procedures (like metastasectomy in our study). This will impede both grant funding and accrual until more compelling response data are obtained or it becomes possible to perform regional therapies outside of the operating room (perhaps easier with suffusion compared with perfusion-based therapies).

Besides the limitations noted above, our work was affected by changes in study personnel, funding mechanisms, and surgical technology. While the overall interval of this report was long, it is really a story of two phases of rapid accrual. The first feasibility pilot study (accrued from 2008–2013) provided long-term safety data relative to the technique of suffusion and, more recently, the surge in accrual for colorectal cancer patients (2022–2025) for whom the treatment of disease has been relatively unchanged, introducing less time bias for the Phase 2 endpoint. Given that it was a novel procedure, multiple protocol amendments were required during technique refinement. This led to diversity in the patient population based on changing inclusion and exclusion criteria. Future machine learning algorithms may be able to help control these effects as our experience increases and the technique becomes more standardized [39,40]. The introduction of more effective systemic therapies (e.g., immune checkpoint inhibitors) also affects the results of regional pharmacotherapeutics. Finally, it is unknown how much the delivery concerns noted above will affect the primary endpoint for the Phase II study. We plan to look for differences in metastatic control based on our measurements of concordance or drug detection.

## 5. Conclusions

Suffusion practiced as both a minimally invasive and open procedure seems safe and is evolving into a more reproducible technique for achieving amplification of drug deliveries. Differential treatment effects were observed in earlier cohorts. Consistency is important now that our highest accruing (CRC) cohort has entered Phase II to determine whether this strategy may suppress the progression of minimal residual disease.

## Figures and Tables

**Table 1:** Patients’ characteristics.

Characteristic	N	Value or Percentage
Age		
All primaries	30	55 ± 12
NSCLC	7	60 ± 10
CRC	16	54 ± 11
Sarcoma (soft tissue)	6	51 ± 16
Breast (triple-negative)	1	64
Female sex	20	67%
Smoking		
Active	0	0%
Former	15	50%
Never	15	50%
Comorbidity		
COPD	7	23.3%
Asthma	2	6.6%
HTN	9	30%
HLD	7	23.3%
CAD	2	6.6%
CKD	1	3.3%
Diverticulosis	5	16.6%
HIV	1	3.3%
Other cancer types	5	16.6%
FEV1 % predicted	30	95.4 ± 20.8
DLCO % predicted	30	83.3 ± 19.1
FVC % predicted	30	104 ± 19.5
6 min walk test % predicted	30	84.5 ± 26.1
Borg dyspnea scale (≥3)	7	(23%)
Suffused side percentage	30	48.3 ± 8.1
Non-suffused side percentage	30	52.2 ± 7.3
Primary cancer treatment		
Chemotherapy	5	16.6%
Chemotherapy + irradiation	3	10%
Chemotherapy + surgery	12	40%
Chemotherapy + surgery + irradiation	5	16.6%
Surgery + irradiation	2	6.6%
Surgery	2	6.6%
Extra-thoracic organs with metastases	7	0.5 ± 0.7
Ipsilateral pulmonary metastases	23	1.3 ± 1.2
Contralateral pulmonary metastases	21	0.3 ± 0.5
Total metastases number	24	1.4 ± 1.3
Pulmonary disease-free interval (months)	23	9.0 ± 15.9
Pre-suffusion pulmonary metastases treatment		
Surgery same side	2	6.6%
Surgery opposite side	3	10%
Irradiation same side	1	3.3%
SBRT same side	2	6.6%
Intrathoracic positive lymph nodes	10	3.3
Lung involvement		
Unilateral	20	66.6%
Bilateral	8	26.7%
Bilateral randomized right	5	62.5%
Bilateral randomized left	3	37.5%
Suffused side		
Right	16	53.3%
Left	14	46.7%
Technique		
VATS	28	93.3%
Open thoracotomy	2	6.7%
Planned surgery		
Wedge only	13	43.3%
Wedge + segmentectomy	2	6.7%
Wedge + lobectomy	5	16.7%
Wedge + lingulectomy	1	3.3%
Segmentectomy	1	3.3%
Lobectomy	3	10%
No tissue removed	5	16.7%
Suffused chemotherapy		
Cisplatin	10	33.3%
Oxaliplatin	14	46.7%
Doxorubicin	4	13.3%
Gemcitabine	2	6.7%

CAD, coronary artery disease; CKD, chronic kidney disease; COPD, chronic obstructive pulmonary disease; CRC, colorectal cancer; DLCO, diffusion capacity for carbon monoxide; FEV1, forced expiratory volume in one second; FVC, forced vital capacity; HIV, human immunodeficiency virus; HLD, Hyperlipidemia; HTN, hypertension; NSCLC, non-small-cell lung cancer; SBRT, Stereotactic Body Radiation Therapy; VATS, video-assisted thoracoscopic surgery.

**Table 2:** Suffusion operative and related data.

Variable	VATS	OPEN	All Cases
VATS for vein control (min)	63 ± 26	102 ± 93	68 ± 25
Vein control to suffusion start (min)	97 ± 42	116 ± 09	99 ± 41
Interventional radiologist time	53 ± 35	11 ± 1	50 ± 35
Suffusion (min) *	30 ± 4	30 ± 0	30 ± 4
Metastasectomy (min)	117 ± 72	274 ± 111	127 ± 83
Total procedural time (min)	399 ± 106	524 ± 123	408 ± 110
Blood aspirated to create suffusate space (mL)	166 ± 78	360 ± 190	179 ± 104
PA (“wedge”) during suffusion (mmHg)	26.7 ± 8.8	33.8 ± 3.3	27.2 ± 8.7
EBL (mL)	79 ± 81	100 ± 71	80 ± 80
Systemic therapy start, lung cancer (weeks)	1.9 ± 2.0	--	1.9 ± 2.0
Chemotherapy restart, if needed (weeks) **	6.8 ± 1.3	11.5 ± 7.5	9.1 ± 5.9
Chest tube removed (day)	1.3 ± 0.2	2.0 ± 1.0	1.5 ± 1.0
Hospital stay (days)	1.5 ± 1.3	6.5 ± 0.5	1.9 ± 1.8

VATS, video-assisted thoracoscopic surgery; EBL, estimated blood loss in milliliters; PA, pulmonary artery. * Two cases were abbreviated (by 5 and 12 min because of occlusion intolerance and catheter dislodgement, respectively). ** Four patients with pulmonary metastases had planned chemotherapy to start after suffusion.

**Table 3:** Long-term results of suffusion based on intent.

#	Dose	Type	Status, Age	Response	DFS (mo)	Comment
Augment Local Control						
1	3.75	Lung	Dead	Complete	59	
2	3.75	Lung	Dead	Complete, Δ	8	Pulmonary recurrence
3	3.75	Lung	Dead	Partial	15	
4	5.625	Lung	Alive, 63	Complete, Δ	151	2nd 1° Breast
6	5.625	Lung	Dead	Mixed, Δ	9	
7	5.625	Lung-B	Dead	Stable	23	
10	7.5	Lung	Alive, 89	Complete, Δ	14	
Suppress Metastases						
5	5.625	Sarcoma	Alive	Complete	165	Leiomyosarcoma
8	5.625	Sarcoma	Dead	Progress	13	MPNST
11	7.5	Breast	Alive, 76	Complete	145	TN

Case 9 did not receive suffusion. # = case number; Δ = indicates that patient had favorable differential response at 2-month assessment in suffused region compared to other systemic disease (Figure 5). 2nd 1° = developed second primary malignancy; Lung-B = multicentric minimally invasive adenocarcinoma; DFS = Disease-Free Survival; dose = mg/m^2^; MPNST = malignant peripheral nerve sheath tumor; TN = triple-negative breast cancer.

## Data Availability

The data presented in this study are available upon request from the corresponding author.

## Supplementary Materials

The following supporting information can be downloaded at: https://www.mdpi.com/article/10.3390/cancers17172880/s1, Supplementary Table S1: Oxaliplatin Patient characteristics Only; Supplementary Table S2: Hospital stay and adverse events; Supplementary Table S3: Suffusion effects on pulmonary function; Supplementary Table S4: 95% confidence levels for values presented in Figure 3 and Figure 4; Video S1: Fluoroscopic video demonstrates open PA flow, PA inflow occlusion alone, and combined with venous snaring (Suffusion). Video S2: Currently preferred PA snaring suffusion variation enabling tissue sampling (also shows metastases localization using preoperative fiducial markers).

## Abbreviations

The following abbreviations are used in this manuscript:

6-MWT	6-min walk test
AEs	Adverse events
ANOVA	Analysis of Variance
CAD	Coronary Artery Disease
CBC	Complete blood count
CMP	Comprehensive metabolic panel
CKD	Chronic Kidney Disease
CRC	Colorectal cancer
CT	Computed tomography
CXR	Chest roentgenograms
DLCO	Diffusion capacity for carbon monoxide
DLT	Dose-limiting toxicity
ECOG	Eastern Cooperative Oncology Group
EKG	Electrocardiography
FEV1	Forced expiratory volume in one second
FVC	Forced vital capacity
HIV	Human immunodeficiency virus
HLD	Hyperlipidemia
HTN	Hypertension
MPNST	Malignant peripheral nerve sheath tumor
MRD	Minimal residual disease
MTD	Maximum tolerated dose
NCI CTCAE v5.0	Common Terminology Criteria for Adverse Events version 5.0
NM	Nuclear medicine
NSCLC	Non-small-cell lung cancer
NSD	Normal systemic dose
PA	Pulmonary artery
PFTs	Pulmonary function tests
PS	Performance status
SBRT	Stereotactic body radiation therapy
SPME	Solid-Phase Micro Extraction
^99^Tc	Technetium-99 macroaggregated albumin
VATS	Video-assisted thoracoscopic surgery
VC	Vital capacity
