# Supplementary material for: Pulmonary Suffusion Refinements for Primary and Secondary Malignancies: Preliminary Analyses of Phase I Safety and Drug Delivery Data
Source: Cancers (Basel). 2025 Sep 2;17(17):2880. doi: 10.3390/cancers17172880 (PMC12427383; doi:10.3390/cancers17172880)
Supplement: Supplementary file 1 [file cancers-17-02880-s001.zip › cancers-3815710-supplementary.pdf]

Supplementary Table S1. Oxaliplatin Patient characteristics Only

Characteristic	N	Value or Percentage
**Oxaliplatin Cases**		
Age		
All primaries		
NSCLC		
CRC	14	55 ± 11
Sarcoma		
Breast (triple negative)		
Female sex	7	50%
Smoking		
Active	0	0%
Former	5	35.7%
Never	9	64.3%
Comorbidity		
COPD	4	28.5%
Asthma	3	21.4%
HTN	4	28.5%
HLD	2	14.3%
CAD	1	7.1%
CKD	1	7.1%
Diverticulosis	3	21.4%
HIV	1	7.1%
Other cancer types	3	21.4%
FEV1 % predicted	14	100.8 ± 18.3
DLCO % predicted	14	68.7 ± 16
FVC % predicted	14	112.9 ± 18.4
6-Minute walk test % predicted	14	74.2 ± 12.5
Borg dyspnea scale (≥3)	4	28.3%
Suffused side percentage	14	49.5 ± 8.5
Non-suffused side percentage	14	50.2 ± 4.1
Primary cancer treatment		
Chemotherapy	0	0%
Chemotherapy + irradiation	1	7.1%
Chemotherapy + surgery	10	71.4%
Chemotherapy + surgery + irradiation	2	14.3%
Surgery + irradiation	0	0%
Surgery	1	7.1%
Extra-thoracic organs with metastases	4	28.5
Ipsilateral pulmonary metastases	14	1.3 ± 0.5
Contralateral pulmonary metastases	9	0.4 ± 0.6
Total metastases number	14	1.8 ± 0.7
Pulmonary disease-free interval (months)	14	14.5 ± 5.2
Pre-suffusion pulmonary metastases treatment		
Surgery same side	1	7.1%

Surgery opposite side	3	21.4%
Irradiation same side	0	0%
SBRT same side	1	7.1%
Intrathoracic positive lymph nodes	4	28.5
Lung involvement		
Unilateral	8	57.1%
Bilateral	6	42.8%
Bilateral randomized right	4	28.6%
Bilateral randomized left	2	14.4%
Suffused side		
Right	8	57.1%
Left	6	42.9%
Technique		
VATS	12	85.7%
Open thoracotomy	2	14.4%
Planned Surgery		
Wedge only	8	57.1%
Wedge + segmentectomy	1	7.1%
Wedge + lobectomy	2	14.4%
Wedge + lingulectomy	1	7.1%
Segmentectomy	1	7.1%
Lobectomy	1	7.1%
No tissue removed	0	0%

COPD, Chronic obstructive pulmonary disease; HTN, hypertension; CAD, coronary artery disease; CKD, chronic kidney disease; DLCO, diffusion capacity for carbon monoxide; FEV1, forced expiratory volume in one second; FVC, forced vital capacity; HIV, human immunodeficiency virus; NSCLC, non-small cell lung cancer; CRC, colorectal cancer; VATS, video-assisted thoracoscopic surgery.

Supplementary Table S2. Hospital stay and adverse events

Drug		Cisplatin	Oxaliplatin	Doxorubicin	Gemcitabine
N		10	14	4	2
Approach		100% VATS	85% VATS	100% VATS	100% VATS
		**5-10% (mg/m²)**	**5-15% (mg/m²)**	**3.75-7.5% (mg/m²)**	**7.5%(mg/m²)**
Dose range tested*		5% (3.75) (3 patients)<br>7.5% (5.625) (6 patients)<br>10% (7.5) (1 patient)	5% (4.25) (3 patients)<br>10% (8.5) (3 patients)<br>15% (12.75) (6 patients)	3.75% (2.8) (3 patients)<br>7.5% (5.625) (1 patient)	7.5% (75) (2 patients)
Histology		NSCLC (7)<br>Sarcoma (2)<br>Breast (1)	CRC (14)	Sarcoma (4)	CRC (2)
Median hospital stay		1	1	1	1
Range**		1-3	1-7	1-2	
Likely suffusion-related dose limiting toxicities (attribution) – 3 patients					
Case #6, grade 3 – (<u>catheter</u>)					
7.5% dose					
Hypotension		1	0	0	0
Troponin elevation		1	0	0	0
Hypoxia		1	0	0	0
Case #12, grade 4 (<u>drug</u>)					
7.5% dose					
Respiratory failure		0	0	1	0
Case #29, grade 3 – (<u>catheter</u>)					
15% dose					
Atrial fibrillation		0	1	0	0
Total	(5)	3	1	1	0
Non-suffusion-related grade 3 toxicities (non DLT) – 6 patients					
Case #1, grade 3, dose 5%					
Pulmonary embolism		1	0	0	0
Case #2, grade 3, dose 5%					
Thrombocytopenia		1	0	0	0
Case #3 & 29, grade 3, dose 5%					
Intestinal obstruction†		1	1	0	0
Case #7, grade 3, dose 7.5%					
Nausea		1	0	0	0
Case #17, grade 3, dose 7.5%					
Mucositis		0	1	0	0
Total	(6)	4	2	0	0

Minor (grade 1 to 2) toxicities (total) including those attributed to metastasectomy					
General					
Pain	(25)	8	7	10	0
Fatigue	(7)	6	1	0	0
Fever/chills	(2)	0	2	0	0
Respiratory					
Cough	(6)	3	2	1	0
Hemoptysis	(4)	4	0	0	0
Pneumothorax	(3)	0	0	2	1
Dyspnea	(7)	2	4	1	0
Hoarseness	(1)	0	0	0	1
Hypoxia	(2)	2	0	0	0
Respiratory others	(2)	0	0	1	1
Cardiovascular					
Arrhythmia	(5)	2	1	2	0
Hypotension	(1)	1	0	0	0
Anemia	(4)	4	0	0	0
Limb edema	(2)	0	1	1	0
Cardiac others	(2)	0	2	0	0
Gastrointestinal					
Taste change	(2)	2	0	0	0
Nausea/vomiting	(5)	2	2	0	1
Constipation	(2)	0	1	1	0
Bloating	(1)	0	1	0	0
Bowel obstruction	(1)	0	1	0	0
Metabolic					
Electrolyte change	(8)	0	6	2	0
Hyperglycemia	(2)	1	1	0	0
Neurologic					
Dizziness	(2)	2	0	0	0
Peripheral neuropathy	(2)	0	2	0	0
Cutaneous					
Pruritus	(1)	0	0	1	0
Skin other	(3)	0	2	1	0
Infectious					
Cellulitis	(1)	1	0	0	0
Enterocolitis	(1)	0	1	0	0
UTI	(1)	0	1	0	0
Procedure others	(8)	0	6	0	2
Total grade 1-2	(111)	40	44	22	5
Total all adverse events	(122)	47	47	23	5
Adverse events per patient	(4.1)	4.7	3.4	5.75	1

CRC, Colorectal cancer; NSCLC, non-small cell lung cancer; DLT, dose-limiting toxicity; UTI, urinary tract infection.

*Percent of a normal systemic dose.
**Range not reported if all cases had same value.
†Bowel obstruction from prior surgical adhesions, not tumor related.

Supplementary Table S3. Suffusion effects on pulmonary function

Parameter	Pre-Suffusion	Predicted from Resection	Observed at 30 Days	Difference
FEV1 (% predicted)	95.4 ± 20.8	84.3 ± 19.5	81.6 ± 14.2	-5.8 ± 18.2
DLCO (% predicted)	83.3 ± 19.1	73.8 ± 18.5	76.8 ± 21.1	1.8 ± 16.4
FVC (% predicted)	104 ± 19.5		89.3 ± 16.9	
Borg dyspnea scale (≥ 3)	7 (23.3)		6 (20)	
6-Minute walk test (% predicted)	84.5 ± 26.1		73.3 ± 21.7	
Suffused side (%)	48.3 ± 8.1	44.9 ± 10.9	38.8 ± 10.5	-6.1 ± 7.1
Non-suffused side (%)	51.6 ± 6.3		60.5 ± 10.9	

FEV1, Forced expiratory volume in one second; DLCO, diffusion capacity for carbon monoxide; FVC, forced vital capacity.

Supplementary Table S4. 95% Confidence Intervals for Pharmacokinetic Data in Figure 4.

	4.25 mg/m2			8.5 mg/m2			12.75 mg/m2		
**Time**	**Mean**	**Upper**	**Lower**	**Mean**	**Upper**	**Lower**	**Mean**	**Upper**	**Lower**
-4	6672	23700	-10355	1606	2354	860	25990	55197	-3217
0	232	384	80	701	1417	-15	770	972	567
15	255	502	8	475	844	106	968	1241	695
60	156	258	54	270	371	168	534	656	412
